# Supplementary material for: Development of a computer-aided design software for the quantitative evaluation of aesthetic damage
Source: PLoS One. 2019 Dec 18;14(12):e0226322. doi: 10.1371/journal.pone.0226322 (PMC6919621; doi:10.1371/journal.pone.0226322)
Supplement: S1 Code site — 1. (ZIP) [file pone.0226322.s001.zip › [EESC_jr][Projeto]Relatorio_07_21/reports/templates_zip/gentelella-master.zip!/gentelella-master/vendors/DateJS/reports/lcov-report/core/parsing_translator.js.html]

Code coverage report for core/parsing\_translator.js


# Code coverage report for core/parsing\_translator.js

## Statements: 79.7% (161 / 202)      Branches: 71.43% (165 / 231)      Functions: 87.5% (28 / 32)      Lines: 79.7% (161 / 202)      Ignored: none

All files » core/ » parsing\_translator.js

```
|  |  |  |
| --- | --- | --- |
| 1 2 3 4 5 6 7 8 9 10 11 12 13 14 15 16 17 18 19 20 21 22 23 24 25 26 27 28 29 30 31 32 33 34 35 36 37 38 39 40 41 42 43 44 45 46 47 48 49 50 51 52 53 54 55 56 57 58 59 60 61 62 63 64 65 66 67 68 69 70 71 72 73 74 75 76 77 78 79 80 81 82 83 84 85 86 87 88 89 90 91 92 93 94 95 96 97 98 99 100 101 102 103 104 105 106 107 108 109 110 111 112 113 114 115 116 117 118 119 120 121 122 123 124 125 126 127 128 129 130 131 132 133 134 135 136 137 138 139 140 141 142 143 144 145 146 147 148 149 150 151 152 153 154 155 156 157 158 159 160 161 162 163 164 165 166 167 168 169 170 171 172 173 174 175 176 177 178 179 180 181 182 183 184 185 186 187 188 189 190 191 192 193 194 195 196 197 198 199 200 201 202 203 204 205 206 207 208 209 210 211 212 213 214 215 216 217 218 219 220 221 222 223 224 225 226 227 228 229 230 231 232 233 234 235 236 237 238 239 240 241 242 243 244 245 246 247 248 249 250 251 252 253 254 255 256 257 258 259 260 261 262 263 264 265 266 267 268 269 270 271 272 273 274 275 276 277 278 279 280 281 282 283 284 285 286 287 288 289 290 291 292 293 294 295 296 297 298 299 300 301 302 303 304 305 306 307 308 309 310 311 312 313 314 315 316 317 318 319 320 321 322 323 324 325 326 327 328 329 330 331 332 333 334 335 336 337 338 339 340 341 342 343 344 345 346 347 348 349 350 351 352 353 354 355 356 357 358 359 360 361 362 | 1 1   1 1327 1327 2853 797   2056 1822       1327     1 577 11   11   11 7 4           1 49 49       49 42     49 49     49 7     49 49     49 49     49 49   49 49       1   528 91   437       2 2 2 2 2 2     108 108 108 108 108 108 108     1 1 1 1   1 1     528   528 525 80   525       35 35 35 1   35       1   12 17       2 3                                 4 11       2 2 2     2         169 169 109 109           154 68 68           50 72 72         230 222               221 221   1 1 1         49 49   49 49 49       49 49   49 2     47 47     47   47       47     530   530   530 2     528 1822 1822     528     528     528 528   528 1 1 1     528               528 1     528 2     528               528             528       528             528 108 108     528 108     528 77     528 528   528       528       528 35     493       493 422   71     493 2 2 2 2   2 2   1     1       2     493 | ``` (function () { 	var $D = Date;   	var flattenAndCompact = function (ax) { 		var rx = []; 		for (var i = 0; i < ax.length; i++) { 			if (ax[i] instanceof Array) { 				rx = rx.concat(flattenAndCompact(ax[i])); 			} else { 				if (ax[i]) { 					rx.push(ax[i]); 				} 			} 		} 		return rx; 	};   	var parseMeridian = function () { 		if (this.meridian && (this.hour \|\| this.hour === 0)) { 			Iif (this.meridian === "a" && this.hour > 11 && Date.Config.strict24hr){ 				throw "Invalid hour and meridian combination"; 			} else Iif (this.meridian === "p" && this.hour < 12 && Date.Config.strict24hr){ 				throw "Invalid hour and meridian combination"; 			} else if (this.meridian === "p" && this.hour < 12) { 				this.hour = this.hour + 12; 			} else Iif (this.meridian === "a" && this.hour === 12) { 				this.hour = 0; 			} 		} 	};   	var setDefaults = function () { 		var now = new Date(); 		Iif ((this.hour \|\| this.minute) && (!this.month && !this.year && !this.day)) { 			this.day = now.getDate(); 		}   		if (!this.year) { 			this.year = now.getFullYear(); 		} 		 		Eif (!this.month && this.month !== 0) { 			this.month = now.getMonth(); 		} 		 		if (!this.day) { 			this.day = 1; 		} 		 		Eif (!this.hour) { 			this.hour = 0; 		} 		 		Eif (!this.minute) { 			this.minute = 0; 		}   		Eif (!this.second) { 			this.second = 0; 		} 		Eif (!this.millisecond) { 			this.millisecond = 0; 		} 	};   	var finishUtils = { 		getToday: function () { 			 if (this.now \|\| "hour minute second".indexOf(this.unit) !== -1) { 				return new Date(); 			} else { 				return $D.today(); 			} 		}, 		setDaysFromWeekday: function (today, orient){ 			var gap; 			orient = orient \|\| 1; 			this.unit = "day"; 			gap = ($D.getDayNumberFromName(this.weekday) - today.getDay()); 			this.days = gap ? ((gap + (orient * 7)) % 7) : (orient * 7); 			return this; 		}, 		setMonthsFromMonth: function (today, orient) { 			var gap; 			orient = orient \|\| 1; 			this.unit = "month"; 			gap = (this.month - today.getMonth()); 			this.months = gap ? ((gap + (orient * 12)) % 12) : (orient * 12); 			this.month = null; 			return this; 		}, 		setDMYFromWeekday: function () { 			var d = Date[this.weekday](); 			this.day = d.getDate(); 			Eif (!this.month) { 				this.month = d.getMonth(); 			} 			this.year = d.getFullYear(); 			return this; 		}, 		setUnitValue: function (orient) { 			Iif (!this.value && this.operator && this.operator !== null && this[this.unit + "s"] && this[this.unit + "s"] !== null) { 				this[this.unit + "s"] = this[this.unit + "s"] + ((this.operator === "add") ? 1 : -1) + (this.value\|\|0) * orient; 			} else if (this[this.unit + "s"] == null \|\| this.operator != null) { 				if (!this.value) { 					this.value = 1; 				} 				this[this.unit + "s"] = this.value * orient; 			} 		}, 		generateDateFromWeeks: function () { 			var weekday = (this.weekday !== undefined) ? this.weekday : "today"; 			var d = Date[weekday]().addWeeks(this.weeks); 			if (this.now) { 				d.setTimeToNow(); 			} 			return d; 		} 	};   	$D.Translator = { 		hour: function (s) { 			return function () { 				this.hour = Number(s); 			}; 		}, 		minute: function (s) { 			return function () { 				this.minute = Number(s); 			}; 		}, 		second: function (s) { 			return function () { 				this.second = Number(s); 			}; 		}, 		/* for ss.s format */ 		secondAndMillisecond: function (s) { 			return function () { 				var mx = s.match(/^([0-5][0-9])\.([0-9]{1,3})/); 				this.second = Number(mx[1]); 				this.millisecond = Number(mx[2]); 			}; 		}, 		meridian: function (s) { 			return function () { 				this.meridian = s.slice(0, 1).toLowerCase(); 			}; 		}, 		timezone: function (s) { 			return function () { 				var n = s.replace(/[^\d\+\-]/g, ""); 				Iif (n.length) { 					this.timezoneOffset = Number(n); 				} else { 					this.timezone = s.toLowerCase(); 				} 			}; 		}, 		day: function (x) { 			var s = x[0]; 			return function () { 				this.day = Number(s.match(/\d+/)[0]); 				Iif (this.day < 1) { 					throw "invalid day"; 				} 			}; 		}, 		month: function (s) { 			return function () { 				this.month = (s.length === 3) ? "jan feb mar apr may jun jul aug sep oct nov dec".indexOf(s)/4 : Number(s) - 1; 				Iif (this.month < 0) { 					throw "invalid month"; 				} 			}; 		}, 		year: function (s) { 			return function () { 				var n = Number(s); 				this.year = ((s.length > 2) ? n : 					(n + (((n + 2000) < Date.CultureInfo.twoDigitYearMax) ? 2000 : 1900))); 			}; 		}, 		rday: function (s) { 			return function () { 				switch (s) { 					case "yesterday": 						this.days = -1; 						break; 					case "tomorrow": 						this.days = 1; 						break; 					case "today": 						this.days = 0; 						break; 					case "now": 						this.days = 0; 						this.now = true; 						break; 				} 			}; 		}, 		finishExact: function (x) { 			var d; 			x = (x instanceof Array) ? x : [x];   			for (var i = 0 ; i < x.length ; i++) { 				Eif (x[i]) { 					x[i].call(this); 				} 			} 			 			setDefaults.call(this); 			parseMeridian.call(this);   			if (this.day > $D.getDaysInMonth(this.year, this.month)) { 				throw new RangeError(this.day + " is not a valid value for days."); 			}   			d = new Date(this.year, this.month, this.day, this.hour, this.minute, this.second, this.millisecond); 			Iif (this.year < 100) { 				d.setFullYear(this.year); // means years less that 100 are process correctly. JS will parse it otherwise as 1900-1999. 			} 			Iif (this.timezone) { 				d.set({ timezone: this.timezone }); 			} else Iif (this.timezoneOffset) { 				d.set({ timezoneOffset: this.timezoneOffset }); 			} 			 			return d; 		}, 		finish: function (x) { 			var today, expression, orient, temp;   			x = (x instanceof Array) ? flattenAndCompact(x) : [ x ];   			if (x.length === 0) { 				return null; 			}   			for (var i = 0 ; i < x.length ; i++) { 				Eif (typeof x[i] === "function") { 					x[i].call(this); 				} 			} 			Iif (this.now && !this.unit && !this.operator) { 				return new Date(); 			} else { 				today = finishUtils.getToday.call(this); 			} 			 			expression = !!(this.days && this.days !== null \|\| this.orient \|\| this.operator); 			orient = ((this.orient === "past" \|\| this.operator === "subtract") ? -1 : 1);   			if (this.month && this.unit === "week") { 				this.value = this.month + 1; 				delete this.month; 				delete this.day; 			}   			Iif ((this.month \|\| this.month === 0) && "year day hour minute second".indexOf(this.unit) !== -1) { 				if (!this.value) { 					this.value = this.month + 1; 				} 				this.month = null; 				expression = true; 			}   			if (!expression && this.weekday && !this.day && !this.days) { 				finishUtils.setDMYFromWeekday.call(this); 			}   			if (expression && this.weekday && this.unit !== "month" && this.unit !== "week") { 				finishUtils.setDaysFromWeekday.call(this, today, orient); 			}   			Iif (this.weekday && this.unit !== "week" && !this.day && !this.days) { 				temp = Date[this.weekday](); 				this.day = temp.getDate(); 				if (temp.getMonth() !== today.getMonth()) { 					this.month = temp.getMonth(); 				} 			}   			Iif (this.month && this.unit === "day" && this.operator) { 				if (!this.value) { 					this.value = (this.month + 1); 				} 				this.month = null; 			}   			Iif (this.value != null && this.month != null && this.year != null) { 				this.day = this.value * 1; 			}   			Iif (this.month && !this.day && this.value) { 				today.set({ day: this.value * 1 }); 				if (!expression) { 					this.day = this.value * 1; 				} 			}   			if (!this.month && this.value && this.unit === "month" && !this.now) { 				this.month = this.value; 				expression = true; 			}   			if (expression && (this.month \|\| this.month === 0) && this.unit !== "year") { 				finishUtils.setMonthsFromMonth.call(this, today, orient); 			}   			if (!this.unit) { 				this.unit = "day"; 			}   			finishUtils.setUnitValue.call(this, orient); 			parseMeridian.call(this); 			 			Iif ((this.month \|\| this.month === 0) && !this.day) { 				this.day = 1; 			}   			Iif (!this.orient && !this.operator && this.unit === "week" && this.value && !this.day && !this.month) { 				return Date.today().setWeek(this.value); 			}   			if (this.unit === "week" && this.weeks && !this.day && !this.month) { 				return finishUtils.generateDateFromWeeks.call(this); 			}   			Iif (expression && this.timezone && this.day && this.days) { 				this.day = this.days; 			}   			if (expression){ 				today.add(this); 			} else { 				today.set(this); 			} 			 			if (this.timezone) { 				this.timezone = this.timezone.toUpperCase(); 				var offset = $D.getTimezoneOffset(this.timezone); 				var timezone; 				Eif (today.hasDaylightSavingTime()) { 					// lets check that we're being sane with timezone setting 					timezone = $D.getTimezoneAbbreviation(offset, today.isDaylightSavingTime()); 					if (timezone !== this.timezone) { 						// bugger, we're in a place where things like EST vs EDT matters. 						Iif (today.isDaylightSavingTime()) { 							today.addHours(-1); 						} else { 							today.addHours(1); 						} 					} 				} 				today.setTimezoneOffset(offset); 			}   			return today; 		} 	}; }()); ``` |
```

Generated by istanbul at Sun Nov 16 2014 12:08:33 GMT-0800 (PST)
